# Modulation of Host Cell Death Signaling Platforms by *Plasmodium falciparum*: Implications for Eryptosis Regulation and Parasite Survival

**DOI:** 10.3390/cells15171603

**Published:** 2026-09-03

**Authors:** Lina Solís-Castillero, Ricardo Correa, Maria Fernanda Alves-Rosa, Carmenza Spadafora

**Affiliations:** 1 Centro de Biología Celular y Molecular de Enfermedades (CBCMe), Instituto de Investigaciones Científicas y Servicios de Alta Tecnología (INDICASAT AIP), City of Knowledge, Panama City ACC92-D5Q28, Panama; lsolis@genetix.com.pa (L.S.-C.); rcorrea@indicasat.org.pa (R.C.); mariaalves@indicasat.org.pa (M.F.A.-R.); 2 Laboratorio Clínico Genetix, Panama City AGC92-MG52D, Panama

**Keywords:** Eryptosis, *Plasmodium falciparum*, Fas (CD95), FasL (CD178L), lipid rafts, erythrocyte membrane remodeling, CASMERs, Regulated Cell Death (RCD), malaria, oxidative stress

## Abstract

Programmed cell death pathways in *Plasmodium falciparum* remain conceptually fragmented. Over decades, researchers have applied metazoan apoptotic, autophagic, and necrotic markers to this deep-branching protozoan, frequently clashing with the reality that the parasite lacks the canonical genetic machinery (such as true caspases or death receptors) found in multicellular eukaryotes. In this work, we shift the focus from the parasite’s disputed intrinsic death machinery to the host–parasite interaction arena: the active manipulation of the host erythrocyte’s autonomous suicide program, eryptosis. The intraerythrocytic development of *P. falciparum* generates profound oxidative stress through hemoglobin digestion and free heme release, driving lipid peroxidation and the accumulation of reactive aldehydes such as 4-hydroxynonenal (4-HNE)—potent signaling molecules that can trigger eryptotic pathways. We integrate existing literature on membrane remodeling, protein export, and lipid raft dynamics to propose a novel Host Protein Sequestration Hypothesis. We suggest that *P. falciparum* evades splenic clearance by actively dismantling the host cell’s surface death signaling platforms—Clusters of Apoptotic Signaling Molecule-Enriched Rafts (CASMERs)—and pulling these host components inward. We suggest that human FAS (CD95) is internalized by the parasite and physically interacts with Plasmodium lipid-raft scaffolding proteins, preventing it from engaging FasL (CD178) that is either expressed on adjacent erythrocytes or presented within the local splenic microenvironment—an interaction that would otherwise precipitate eryptotic signaling. This perspective offers a fundamentally fresh conceptual framework for understanding malaria survival strategies and highlights a vulnerable, non-canonical therapeutic target.

## 1. Introduction

Malaria continues to be a major public health crisis globally, driving high rates of illness and death, with the heaviest burden falling on sub-Saharan Africa. Among the species infecting humans, *P. falciparum* is the primary cause of the most severe and fatal disease manifestations. While combination therapies featuring artemisinin and other drugs have been deployed to combat the infection, the rising prevalence of drug-resistant parasite strains and mosquitoes resistant to insecticides threatens these global control and eradication initiatives) [1,2,3,4].

To combat the parasite during its clinical stages, a number of antimalarial therapies are designed to disrupt vital metabolic processes and organelle functions. For example, some drugs act against the parasite’s mitochondrial electron transport chain, target the specialized apicoplast organelle responsible for crucial biosynthesis, or interfere with heme detoxification and the parasite’s ability to manage oxidative stress [5,6,7,8].

A major consequence of *P. falciparum* intraerythrocytic metabolism is the generation of massive oxidative stress. The parasite digests up to 75% of the host cell hemoglobin within the digestive vacuole, releasing free heme—a potent pro-oxidant that catalyzes the formation of reactive oxygen species (ROS) through Fenton chemistry [9,10]. This oxidative burden is further amplified by the parasite’s own metabolic activity, including mitochondrial respiration and the synthesis of redox-active compounds [11]. The resultant ROS attack polyunsaturated fatty acids within the erythrocyte membrane, initiating a chain of lipid peroxidation that ultimately generates highly reactive electrophilic aldehydes, most notably 4-hydroxynonenal (4-HNE) [12,13]. 4-HNE is not merely a passive end-product of oxidative damage; it functions as a second messenger that covalently modifies proteins via Michael addition to cysteine, histidine, and lysine residues, thereby altering enzyme activities, ion channel function, and signaling cascades [14,15]. In erythrocytes, 4-HNE has been shown to impair the activity of key membrane transporters, disrupt the cytoskeletal network, and promote phosphatidylserine (PS) externalization—hallmarks of eryptosis [16,17]. Importantly, 4-HNE can also modulate lipid raft organization by altering membrane fluidity and cholesterol distribution, potentially influencing the clustering of death receptors such as Fas within CASMER platforms [18]. Thus, the interplay between parasite-induced oxidative stress, 4-HNE generation, and membrane remodeling represents a critical axis that may determine whether an infected erythrocyte undergoes premature clearance or survives to support parasite replication.

Environmental pressures and stressors such as exposure to antimalarial drugs, febrile host temperatures, starvation, and oxidative stress can trigger Regulated Cell Death (RCD) in *P. falciparum*. During these events, the parasite frequently exhibits morphological signs that closely resemble apoptosis in metazoans, including DNA fragmentation, cellular shrinkage, exposure of phosphatidylserine on the membrane, and a drop in mitochondrial membrane potential [19,20]. This indicates that the malaria parasite possesses key biomolecular structures enabling it to undergo events classically considered to be apoptosis in higher organisms [21,22,23,24]. Despite exhibiting these characteristic features of cell death, there is currently a lack of conclusive evidence showing that *P. falciparum* possesses the classical caspase-driven molecular machinery responsible for executing regulated apoptosis like in metazoans.

To survive and replicate within a mature erythrocyte—a cell devoid of organelles for protein synthesis and trafficking—*P. falciparum* actively imports and exports hundreds of specialized effector proteins. This extensive host cell modification represents a targeted, parasite-driven reprogramming of the erythrocyte environment rather than a passive consequence of infection [25,26,27]. Over the past three decades, comprehensive research into the human erythrocyte membrane has revealed it to be a highly specialized composite structure rather than a simple envelope. The membrane consists of a lipid bilayer containing roughly equal proportions of cholesterol and phospholipids that is tethered to an underlying two-dimensional elastic protein skeleton via specific transmembrane proteins. This underlying skeletal network is highly complex and is constructed from a specific list of interacting proteins, primarily α- and β-spectrin, actin, protein 4.1R, adducin, dematin, tropomyosin, and tropomodulin [28,29]. Furthermore, contrary to the misconception that this structure contains only a few proteins, mass spectrometry and proteomic analyses have cataloged 751 unique proteins in the human erythrocyte [30]. This vast proteome includes over 50 well-characterized transmembrane proteins governing vital transport, adhesion, and structural functions, such as band 3, glycophorins, and aquaporins, along with hundreds of other associated structural and functional proteins. Crucially, it is this exact sophisticated architectural framework that *Plasmodium falciparum* must systematically dismantle and actively subjugate following invasion. During its 48 h intraerythrocytic developmental cycle, the malaria parasite exports more than 400 of its own specialized effector proteins directly into the host cell cytoplasm. A substantial fraction of these parasite-derived proteins is specifically designed to target and physically interface with the host’s native membrane skeleton and integral transmembrane receptors [28,29,30,31].

## 2. RCD in *P. falciparum*: From Canonical Apoptosis to Alternative Stress-Response Pathways

**Figure 1:**
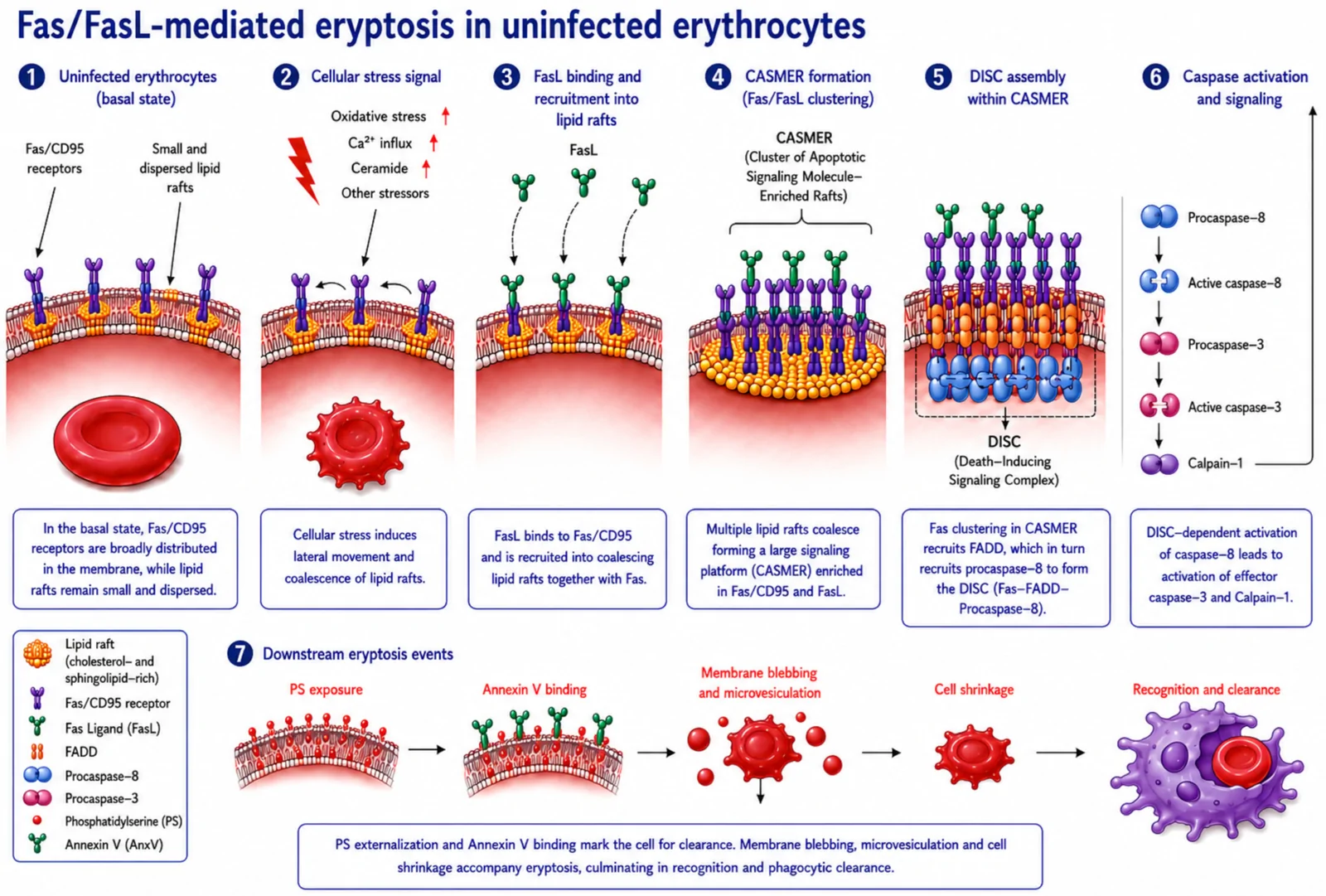
**Fas/FasL-mediated eryptosis pathway in uninfected erythrocytes.** (1) Under basal conditions, Fas/CD95 receptors are broadly distributed across the erythrocyte membrane, while lipid rafts remain small and dispersed. (2) Cellular stress—including oxidative stress, increased Ca^2+^ influx, and ceramide accumulation—promotes the lateral redistribution of Fas/CD95 and the coalescence of lipid rafts. (3) Fas ligand (FasL) binds Fas/CD95 and is recruited with the receptor into the coalescing membrane domains. (4) The resulting Fas/FasL clustering generates a larger signaling platform termed CASMER (cluster of apoptotic signaling molecule-enriched rafts). (5) Fas clustering within CASMER recruits FADD and procaspase-8, leading to formation of the death-inducing signaling complex (DISC). (6) DISC assembly promotes caspase-8 activation, followed by activation of the effector caspase-3 and calpain-1. (7) These signaling events culminate in phosphatidylserine (PS) externalization, Annexin V binding, membrane blebbing and microvesiculation, cell shrinkage, and recognition and clearance of the eryptotic erythrocyte by phagocytic cells. This schematic represents the proposed sequence of events and does not imply that every signaling component has been fully validated in erythrocyte. The figure was created using ChatGPT GPT-5.6 Sol) and subsequently reviewed and edited by the authors.

In metazoans, RCD is a highly orchestrated biological process executed through specific signaling cascades (Figure 1). The primary modalities of RCD include apoptosis, pyroptosis, necroptosis, and autophagy. Apoptosis represents a generally non-inflammatory process orchestrated by the caspase family, marked by early preservation of the cell membrane, cellular condensation, and chromatin clumping. In contrast, pyroptosis is a highly inflammatory mechanism where caspases cleave gasdermin proteins (such as GSDMD) to create pores in the plasma membrane, ultimately causing the cell to swell and burst. Necroptosis is a caspase-independent, regulated form of cell death that shares morphological similarities with necrosis (such as cellular swelling and rupture) but is tightly controlled by kinases such as RIPK1, RIPK3, and MLKL. Finally, autophagy generally supports cellular metabolism through the lysosomal degradation of engulfed contents, but it can also operate in tandem with apoptosis as an alternative cell death mechanism [32,33,34].

Two main pathways of RCD are particularly relevant in metazoans: canonical apoptosis, which engages either an intrinsic (mitochondrial) or extrinsic (receptor-mediated) pathway controlled by caspases; and RCD pathways triggered by DNA damage, which are governed by key proteins like the BCL-2 family members, caspases, and p53 [35,36]. Importantly, recent studies indicate that fundamental features of these processes are also present in unicellular organisms [37,38].

Although *P. falciparum* lacks the classical apoptotic pathways seen in higher eukaryotes, the parasite exhibits an apoptosis-like death under stressful conditions. During these events, the parasite undergoes morphological and biochemical changes, including chromatin condensation, DNA fragmentation, mitochondrial depolarization, exposure of phosphatidylserine on the outer plasma membrane, and cellular shrinkage [19,20,39].

Among the most potent inducers of this apoptosis-like death in *P. falciparum* is oxidative stress, which arises from the parasite’s catabolism of host hemoglobin and the subsequent release of free heme and iron [10]. The resulting ROS not only inflict direct damage to parasite nucleic acids, proteins, and lipids but also trigger a cascade of lipid peroxidation that generates 4-HNE and other reactive aldehydes [12]. In *P. falciparum*, 4-HNE has been detected in parasitized erythrocytes and correlates with markers of oxidative injury [40]. Although the direct effects of 4-HNE on parasite RCD pathways remain incompletely characterized, its capacity to modify parasite proteins—particularly those involved in redox defense and metabolic regulation—suggests that it may contribute to the stress signals that push the parasite toward an apoptosis-like fate [9,15]. Furthermore, 4-HNE adduction to erythrocyte membrane proteins can destabilize the host cytoskeleton, increase membrane permeability, and promote PS exposure, all of which are features that overlap with both parasite RCD and host eryptosis [16,17]. This convergence underscores the difficulty of distinguishing parasite-intrinsic death from host cell demise, as oxidative stress and lipid peroxidation products simultaneously affect both compartments. Crucially, this form of cell death in *P. falciparum* operates independently of the classical caspases and death receptor-mediated pathways characteristic of metazoans [21,22,23,41]. In unicellular organisms, apoptosis-like processes are theorized to be an evolutionary adaptation allowing for the elimination of stressed subpopulations. By sacrificing a portion of the population, the parasite favors the survival of the remaining cells, ensuring successful transmission to new hosts by avoiding excessive tissue damage or the premature death of the host [42,43]. Consequently, this phenomenon has been interpreted through the lens of kin selection and parasite population regulation [44,45,46].

In contrast to its lack of canonical apoptosis-related genes, *P. falciparum* has partial or even fully functional homologs of stress response-related genes found in higher eukaryotes [47]. As an example, it was previously suggested that *P. falciparum* lacks an ortholog of p53 [48], a protein that mediates numerous aspects of the stress response in metazoans. However, recent genomic analyses have actually identified a putative p53 homolog within the parasite [49]. In addition, the parasite contains two other putative stress response regulators, PfMDM2 and PfSWIB, which are each predicted to contain a SWIB/MDM2 domain. This domain is a conserved protein interaction structure commonly found in chromatin-remodeling and transcription-regulating proteins of higher eukaryotes, and its presence in PfMDM2 and PfSWIB suggests that it may be involved in a stage-specific heat-stress response pathway in *P. falciparum* [49,50]. Additionally, the parasite relies on a separate protein known as DNA damage-inducible protein 1 (PfDDI1) to regulate stress responses and protein degradation through the ubiquitin-proteasome system [51]. Despite the presence of these various stress response elements, it has been suggested that even if a parasite dies through an RCD process, it does not necessarily mean that the exact same set of conserved molecular pathways is triggered as in other eukaryotes [23,51,52,53].

It is important to note that there are limitations in the Interpretation of cell death in malaria-stricken organisms. We are caught in a blind spot trying to decipher a chaotic biological triad: the immune system’s attempt to eliminate the threat via external signals like Fas/FasL, the infected red blood cell’s primitive suicide mechanism (eryptosis) trying to abort its deadly cargo, and the hidden stress-induced RCD machinery of the parasite itself utilizing uncharacterized routes. Attempting to experimentally measure or isolate these pathways is very challenging. Because these pathways overlap within the same microenvironment, traditional assays cannot definitively tease apart whether a death signal is due to the host’s defense, the erythrocyte’s collapse, or the parasite’s internal distress.

Thus, interpreting *P. falciparum* cell death relying on assays optimized for mammalian systems could be misleading [21]. TUNEL assays are not exclusive to apoptosis and can yield false positives by labeling DNA breaks associated with active transcription, genomic repair, or necrosis [54,55]. Fluorogenic caspase substrates cross-react extensively with other parasitic cysteine proteases (such as calpains or cathepsins), giving a false impression of caspase activity [56,57,58]. Annexin V binding is highly ambiguous in this context; because the parasite is encapsulated, a positive signal often reflects stress-induced remodeling or eryptosis of the host erythrocyte membrane rather than an intrinsic apoptotic process within the parasite itself [59,60,61] (Figure 1). As for the activity of metacaspases, caspase probes are standard commercial fluorogenic substrates and pan-inhibitors (e.g., CaspACE, z-VAD-fmk) that target aspartate specificity. Because parasite metacaspases cleave basic residues, positive signals from these probes likely reflect cross-reactivity with non-caspase parasitic cysteine proteases like calpains or cathepsins [21,47,57,62]. The summary of what is known of RCD in *P. falciparum* is shown in Table 1.

## 3. Host Membrane Remodeling and Host-Derived Death Signaling in *P. falciparum*

The survival of *P. falciparum* during its asexual blood stage fundamentally depends on its ability to successfully complete replication within the host red blood cell. To evade splenic clearance and phagocytic recognition by macrophages of the spleen and liver, the parasite extensively remodels the infected erythrocyte, altering its membrane permeability, cytoskeletal architecture, mechanical properties, and characteristic discoid shape. These modifications also promote cytoadherence of infected erythrocytes to the vascular endothelium, thereby reducing their passage through the spleen [31,63].

Despite these parasite-induced adaptations to prolong erythrocyte survival, infection can also activate host defense mechanisms aimed at eliminating infected erythrocytes before parasite replication is completed. Although mature human erythrocytes lack internal organelles and active transcriptional machinery, they retain innate programmed cell death pathways [64]. These mechanisms enable the red blood cell to undergo an apoptosis-like demise, eryptosis, when exposed to oxidative stress, cellular aging, metabolic impairment, elevated intracellular calcium levels, various hematological disorders, or direct parasitic infection by pathogens such as *P. falciparum* [60,61,65]. The induction of eryptosis prompts the translocation of phosphatidylserine to the outer leaflet of the plasma membrane. This externalization acts as an “eat-me” signal, promoting recognition and phagocytosis by splenic and hepatic macrophages, thereby facilitating the clearance of damaged or infected erythrocytes. Consequently, eryptosis serves as an important host defense mechanism by limiting parasite survival and preventing the progression of infection [60,65] (Figure 1).

To execute this extensive remodeling, *P. falciparum* exports hundreds of specialized effector proteins that collectively transform the host cell’s rigidity, permeability, cytoadherence, and membrane composition [26]. This massive protein export relies on highly sophisticated trafficking networks. Central to this process are the Plasmodium PTEX, parasite-induced vesicular intermediates, and distinct membranous sorting compartments known as Maurer’s clefts, which are strategically distributed throughout the host cytoplasm [26,66,67,68,69]. Once delivered, numerous exported parasite proteins directly interface with critical erythrocyte cytoskeletal components, including actin, ankyrin, spectrin, Band 3, and Band 4.1. These interactions extensively remodel the host cell, enabling the parasite to maintain intracellular survival, modify erythrocyte mechanical properties, and promote cytoadherence, all of which are critical for parasite development and immune evasion [26]. Consequently, the infected erythrocyte becomes a highly dynamic and extensively remodeled cellular environment rather than a passive host cell.

Current literature also explores additional potential mechanisms driving apoptosis-like responses in *P. falciparum*, particularly the concept of molecular or functional mimicry. Through this evolutionary strategy, structurally distinct parasite proteins acquire functional properties that closely resemble those of native host molecules. In *P. falciparum*, this phenomenon is indirectly observed among the highly variant protein families exported to the host cell surface, particularly PfEMP1, RIFIN, and STEVOR. For instance, structural studies have revealed that the PfEMP1 family can precisely mimic the binding features of native host ligands to interact with the endothelial protein C receptor (EPCR), maintaining host receptor engagement despite extreme parasite sequence diversity [70]. These membrane-localized parasite proteins efficiently interact with human endothelial and immune receptors, allowing the parasite to actively modulate the host’s immune response, promote severe tissue sequestration, facilitate cellular adhesion, and ultimately evade splenic clearance [70,71,72,73,74]. Although direct evidence linking molecular mimicry to the modulation of host apoptotic pathways in *P. falciparum* is currently lacking, increasing evidence from mammalian systems highlights the importance of the spatial organization of death receptors and signaling molecules in regulating apoptosis. Understanding these mechanisms provides a useful conceptual framework for exploring how parasite-derived proteins may interfere with host cell death signaling during infection.

In human research, particularly within cancer research, investigations have demonstrated that the extrinsic pathway of FAS-mediated apoptosis frequently involves the translocation of FAS and numerous downstream signaling proteins into lipid rafts [18,75,76]. This targeted redistribution facilitates the assembly of the death-inducing signaling complex (DISC) by generating localized, high-concentration platforms that coordinate the rapid activation of apoptotic signals. These specific cholesterol-rich microdomains have been formally designated as CASMERs (clusters of apoptotic signaling molecule-enriched rafts). CASMERs function as dynamic, proapoptotic scaffolds that concentrate death receptors, adaptor molecules, and executioner caspases, thereby heavily amplifying the apoptotic cascade through the precise spatial compartmentalization of the DISC machinery [18]. Consequently, the spatial clustering of these crucial signaling molecules within lipid rafts is increasingly recognized as a fundamental regulatory mechanism for efficiently executing death-receptor pathways.

During its intraerythrocytic development, *P. falciparum* extensively restructures the host erythrocyte’s membrane lipid organization [25,77,78,79]. This profound remodeling alters cholesterol distribution, disrupts normal phospholipid asymmetry, increases overall membrane fluidity, and specifically modifies lipid raft microdomains. These specific cholesterol- and sphingolipid-enriched rafts naturally harbor critical membrane-organizing proteins belonging to the stomatin/prohibitin/flotillin/HflK/C (SPFH) family, such as stomatin, flotillins, and prohibitins. In healthy cells, these specialized proteins participate in the organization of signaling complexes, membrane trafficking, and generalized responses to cellular stress [80]. Because mature human erythrocytes are terminally differentiated and possess negligible endogenous capacities for de novo lipid biosynthesis or vesicular trafficking, these extensive membrane alterations strongly point to an active, parasite-directed reconstruction of the host cell’s structural architecture. Indeed, recent structural analyses reveal that this active manipulation begins at the precise moment of host cell entry, when the parasite’s moving junction complex (comprising AMA1 and RON proteins) inserts amphipathic helices directly into the inner leaflet of the host membrane to severely disrupt local lipid packing and drive membrane remodeling [81].

Several studies indicate that *P. falciparum* does not solely rely on the export of its own parasite-derived proteins into the erythrocyte cytoplasm. Instead, the parasite actively hijacks and redistributes native host membrane components to facilitate its intracellular survival and secure nutrient acquisition [79]. This phenomenon of host protein hijacking raises the compelling possibility that endogenous host signaling molecules, specifically those associated with stress responses and eryptosis, could be actively redistributed into parasite-induced membrane microdomains during the course of infection.

During an active malaria infection, native host signaling components alone cannot account for the sheer scale of the observed membrane remodeling, confirming that parasite-derived effector proteins actively participate in shaping this highly altered intraerythrocytic environment [79]. Infected erythrocytes are subjected to profound structural and biochemical perturbations, characterized by severe oxidative stress, ionic gradients, disrupted phospholipid asymmetry, enhanced membrane fluidity, extensive cytoskeletal destabilization, and the genesis of parasite-induced membranous compartments [25,26]. Furthermore, there is evidence that *P. falciparum* internalizes components of the erythrocyte membrane such as cholesterol and GPI-anchored proteins [82].

The potential hijacking of these domains is particularly relevant in the context of eryptosis. Under specific pathological conditions that trigger this RCD, such as profound oxidative stress and elevated intracellular calcium levels, key protein mediators of extrinsic eryptosis, including FAS, caspase-8, and caspase-3, rapidly translocate into the erythrocyte’s lipid rafts [64,83]. Once concentrated within these microdomains, these molecules contribute to the organization and amplification of pro-eryptotic signaling cascades. This compartmentalized activation is typically accompanied by the externalization of phosphatidylserine (PS) to the outer leaflet of the plasma membrane, robust caspase cleavage, and progressive structural remodeling of the host cell [84].

Notably, recent comprehensive analyses clarify that because mature erythrocytes lose their internal organelles during erythropoiesis, their RCD machinery is highly streamlined compared to that of nucleated cells. Rather than functioning as essential initiators of eryptosis, the retention and precise spatial compartmentalization of FAS and caspase-8 within these lipid microdomains are now thought to regulate how stress signals are integrated within the erythrocyte. The retention and precise spatial compartmentalization of caspase-8 and FAS within these lipid microdomains are now understood to function as a highly sensitive molecular switch. Within this streamlined regulatory network, raft-associated caspase-8 operates at the crossroads of cell fate decisions, actively mediating the necessary crosstalk between non-lytic, anti-inflammatory eryptosis and lytic, pro-inflammatory erythronecroptosis [64].

Thus, if *P. falciparum* actively hijacks and redistributes these specific raft-associated signaling platforms, the parasite could fundamentally manipulate this cellular switch. Such a targeted intervention would not only allow the parasite to delay premature eryptotic clearance by macrophages, but also prevent the infected host cell from undergoing a lytic necroptotic death that would prematurely terminate the parasite’s intraerythrocytic replication cycle.

Collectively, these observations support the hypothesis that *P. falciparum* may directly interfere with the compartmentalization, redistribution, and ultimate signaling activity of host-derived pro-eryptotic molecules residing within these specialized lipid raft microdomains. In this specific biochemical context, it is highly plausible that mechanisms conceptually similar to CASMER formation emerge during active malaria infection. This phenomenon would likely occur through the targeted clustering or re-localization of native erythrocyte death-signaling components within host lipid rafts. By actively reorganizing these specific microdomains, the parasite could effectively alter eryptotic signaling thresholds, thereby significantly delaying the premature clearance of the infected red blood cell. Such deliberate molecular modulation would provide a distinct survival advantage, granting the infected erythrocyte the necessary longevity to successfully complete the parasite’s 48 h intraerythrocytic replication cycle. Concurrently, this crucial delay in host cell death heavily contributes to the parasite’s broader strategies for host adaptation and immune evasion [60,61].

The organization and function of lipid rafts—and by extension CASMERs—are exquisitely sensitive to oxidative stress and lipid peroxidation. 4-HNE, generated during oxidative attack on membrane phospholipids, incorporates into the lipid bilayer and profoundly alters its biophysical properties [12,15]. At low concentrations, 4-HNE can act as a signaling molecule, promoting the clustering of lipid rafts and the recruitment of death receptors such as Fas into these microdomains, thereby sensitizing cells to apoptotic stimuli [14]. However, at higher concentrations, 4-HNE induces extensive cross-linking of membrane proteins, disrupts lipid–protein interactions, and promotes the shedding of membrane vesicles, which may remove death receptors from the cell surface [17,85]. In the context of *P. falciparum* infection, the parasite-induced oxidative environment likely generates a complex and dynamic pattern of 4-HNE accumulation within the host erythrocyte membrane. This could have dual effects: (i) initially, 4-HNE may promote CASMER formation and sensitize the infected erythrocyte to eryptosis, creating a host defense mechanism; (ii) over time, sustained 4-HNE production could lead to extensive membrane damage and vesiculation, potentially removing Fas and other signaling molecules from the surface, thereby inadvertently aiding the parasite’s evasion of immune clearance. The parasite may also actively modulate 4-HNE levels by importing host antioxidant enzymes such as peroxiredoxin-2 [86] or by exporting proteins that scavenge reactive aldehydes. Thus, the balance between 4-HNE production and detoxification may represent a critical determinant of infected erythrocyte fate.

Taken together, the current body of evidence strongly indicates that *P. falciparum* employs a highly sophisticated, multifactorial strategy to control erythrocyte physiology. This comprehensive approach involves massive export of parasite proteins, extensive structural remodeling of membranes, dynamic reorganization of lipid microdomains, selective hijacking of host signaling components, and potential functional mimicry. Beyond simply facilitating initial host cell invasion and driving clinical virulence, these integrated processes likely serve a critical regulatory role in modulating host eryptotic signaling networks. By actively subverting these innate cell death mechanisms, the parasite ensures that it can transiently evade premature host cell elimination, thereby optimizing the intracellular environment for its own successful survival and replication [60,61].

## 4. Eryptosis and Fas Signaling

While *P. falciparum* actively subverts host cell survival pathways to prevent premature splenic clearance, understanding this manipulation requires a closer look at how the erythrocyte’s suicidal machinery is regulated across its lifespan. Eryptosis is triggered by a variety of stressors, among which oxidative stress plays a prominent role. The exposure of erythrocytes to ROS promotes the peroxidation of membrane lipids, generating 4-HNE and other reactive aldehydes that serve as both damaging agents and signaling molecules [16,17]. 4-HNE has been shown to directly stimulate eryptosis by activating the Gardos channel (Ca^2+^-activated K^+^ channel), leading to cell shrinkage and PS exposure, as well as by inhibiting the aminophospholipid translocase that normally maintains PS asymmetry [16,87]. Moreover, 4-HNE can modify the erythrocyte cytoskeleton, promoting membrane destabilization and vesiculation [85]. In the context of *P. falciparum* infection, the parasite-induced oxidative burst generates a continuous supply of ROS and 4-HNE within the host erythrocyte, creating a pro-eryptotic milieu that the parasite must actively counteract [10,60]. This sets the stage for a dynamic interplay: the parasite’s metabolic activity generates the very oxidative signals that threaten its host cell, while the parasite must simultaneously deploy antioxidant and membrane-stabilizing strategies to prevent premature eryptosis. During the transition from the bone marrow to the peripheral circulation, developing red blood cells must strictly downregulate surface Fas (CD95) to reduce their sensitivity to eryptotic signaling. In their earliest developmental stages, nucleated erythroblasts express high baseline levels of CD95 on their plasma membranes. This presence is physiologically necessary within the bone marrow’s erythroblastic islands, where Fas/FasL-mediated apoptosis acts as a critical negative feedback loop to control the overproduction of red blood cells and eliminate defective progenitors [88,89].

However, once the cell commits to entering the bloodstream as a reticulocyte, retaining this death receptor becomes a severe survival liability. To reduce this vulnerability, reticulocytes undergo extensive membrane remodeling and selectively eliminate proteins that are no longer required during terminal maturation. This process involves coordinated changes in endosomal trafficking, autophagy, and extracellular vesicle release rather than a single degradation pathway [90,91,92]. During reticulocyte maturation, membrane proteins can be internalized into endosomal compartments, which may develop into multivesicular bodies (MVBs). These MVBs can subsequently fuse with the plasma membrane, releasing intraluminal vesicles as exosomes, thereby contributing to the selective removal of specific membrane components. Because the reticulocyte is actively dismantling its internal lysosomal degradation pathways, it utilizes an alternative disposal mechanism: MVBs traffic to the cell periphery and fuse with the plasma membrane, releasing the Fas receptors into the extracellular space in microvesicles. This exosomal shedding pathway, originally characterized by Johnstone et al. [93] for the clearance of transferrin receptors, provides a general mechanism by which reticulocytes eliminate selected membrane proteins during maturation. Whether Fas/CD95 is efficiently removed through this pathway remains an area of investigation.

Unlike nucleated cells, which dynamically regulate their sensitivity to apoptotic signals by internalizing the Fas receptor within a cytoplasmic Golgi reserve or internalizing it into endosomes, mature erythrocytes exhibit a fundamentally different structural dynamic. Following nuclear extrusion and organelle loss during maturation, erythrocytes lose the capacity to maintain an internal, cytoplasmic pool of Fas [64]. Consequently, the remaining receptor is restricted entirely to the plasma membrane. In this exposed location, surface-bound Fas functions as a critical sentinel; upon encountering severe oxidative stress, energy depletion, or calcium influx, it can trigger eryptosis—the specialized form of suicidal cell death that ensures the safe and silent removal of defective red blood cells from circulation before they undergo catastrophic hemolysis [84].

Because mature erythrocytes lack the endolysosomal machinery required to internalize and degrade membrane proteins, they must employ an outward-facing mechanism to manage surface receptor dynamics and clear damaged cellular components. During physiological aging or in response to redox stress, erythrocytes undergo extensive membrane vesiculation, pinching off small fragments of their plasma membrane to form extracellular microvesicles. This shedding process effectively extrudes membrane-bound proteins, including the Fas receptor, into the extracellular space rather than pulling them inward into the cytoplasm. As highlighted in the broader context of redox biology and erythrocyte storage [85,94], the release of these membrane-derived microvesicles serves as a vital adaptive mechanism. It allows the stressed erythrocyte to selectively dispose of altered, damaged, or signaling-effective membrane architecture, thereby preserving the structural integrity of the circulating cell for as long as possible [84].

Despite the progressive reduction in apoptotic receptors during erythrocyte maturation, Fas remains functionally relevant in pathological contexts. This apparent paradox may reflect the fact that erythrocyte membrane remodeling is not equivalent to complete receptor elimination. Instead, mature erythrocytes retain a minimal repertoire of signaling molecules that can act as damage-associated recognition platforms [64]. During *P. falciparum* infection, parasite-induced alterations in membrane architecture, including increased rigidity [95,96], cytoskeletal remodeling [97], and oxidative stress [98], may disrupt normal vesicular shedding processes and modify the accessibility or distribution of Fas on the infected erythrocyte surface. Such changes could create a permissive environment for FasL-expressing immune cells, including NK cells and eosinophils, to recognize and eliminate parasitized erythrocytes.

The work by Mavoungou et al. (2003) [99] provided critical in vitro evidence that the innate immune system actively utilizes the Fas pathway to recognize and destroy *P. falciparum*-infected erythrocytes. The study demonstrated that human Natural Killer (NK) cells can directly mediate the cytolysis of parasitized red blood cells, effectively inhibiting the replication of the parasite’s asexual blood stages. Rather than relying solely on macrophage-mediated phagocytosis, this direct cytotoxic interaction established that the Fas death receptor serves as a functional target on the heavily modified membrane of the infected host cell. The dependency of this cytolytic process on the Fas/FasL axis was proven through targeted inhibition experiments. When the researchers pre-treated the in vitro co-cultures with an antagonistic anti-CD95 monoclonal antibody or introduced a human Fas-Fc soluble fusion protein to neutralize the ligand, the NK cell-mediated inhibition of parasite growth was completely abrogated. This finding confirmed that the specific engagement of the Fas receptor on the erythrocyte surface by Fas Ligand-expressing NK cells is a mandatory molecular checkpoint required to initiate the destruction of the parasitized cell.

Furthermore, the study above linked the initial Fas engagement to the deployment of downstream cytolytic effector molecules. The researchers observed that neutralizing the Fas/FasL interaction also halted the required activity of granzyme B and perforin. This suggests a coordinated, sequential mechanism in which Fas engagement serves as the primary recognition and activation signal that instructs the NK cell to release its cytotoxic granules directly into the immunological synapse, ensuring the structural breakdown of the parasitized erythrocyte.

Beyond NK cells, other innate immune effectors like eosinophils also express functional FasL and secrete potent cytotoxic proteins (such as Eosinophil Cationic Protein) [100]. Because eosinophil degranulation and recruitment are active during malaria infections, their ability to engage exposed surface death receptors on modified erythrocytes provides an additional layer of immune pressure. This further highlights how residual CD95 on the host membrane acts as a broad target for innate immune surveillance, reinforcing the selective advantage for the parasite to actively sequester these platforms.

Therefore, the findings by Mavoungou and colleagues [99] repositioned CD95 from being a mere structural remnant on the mature red blood cell to an active immunological vulnerability for the intraerythrocytic parasite. By exploiting the baseline Fas receptors remaining on the host membrane, the innate immune system can trigger the premature cytolysis of the infected erythrocyte and successfully halt the replication cycle, thus limiting the exponential expansion of *P. falciparum* parasitemia.

## 5. The Host Protein Sequestration Hypothesis: A New Framework for Parasite Survival

We propose that *P. falciparum* actively dismantles host cell surface death-signaling platforms (CASMERs) by sequestering Fas into intracellular compartments. This sequestration is mediated by physical interactions with parasite-encoded lipid-raft scaffolding proteins. Consequently, the infected erythrocyte becomes refractory to extrinsic eryptosis signals, allowing the parasite to complete its intraerythrocytic replication cycle.

An intriguing implication of this hypothesis is that Fas sequestration may represent more than a mechanism to prevent eryptosis. The extensive remodeling of the erythrocyte membrane induced by *P. falciparum* could, in principle, increase the likelihood that surface-exposed Fas molecules become engaged by FasL expressed by activated immune cells. If this occurs in vivo, the parasite would be subjected to an additional layer of immune pressure beyond the intrinsic stress imposed by the process of infecting a host’s foreign cell.

From this perspective, sequestration of Fas away from the plasma membrane may have evolved as a strategy for immune evasion, preventing activation of an extrinsic death pathway that could otherwise arise from the interplay between parasite-induced membrane remodeling and host immune surveillance. This scenario is consistent with a process of host–parasite coevolution, whereby the appearance of Fas on the surface of infected erythrocytes as part of a host defensive response may have imposed a selective pressure on the parasite. The internalization of Fas from the plasma membrane could therefore represent an adaptive countermeasure that limits Fas-mediated signaling and helps preserve parasite survival.

**Figure 2:**
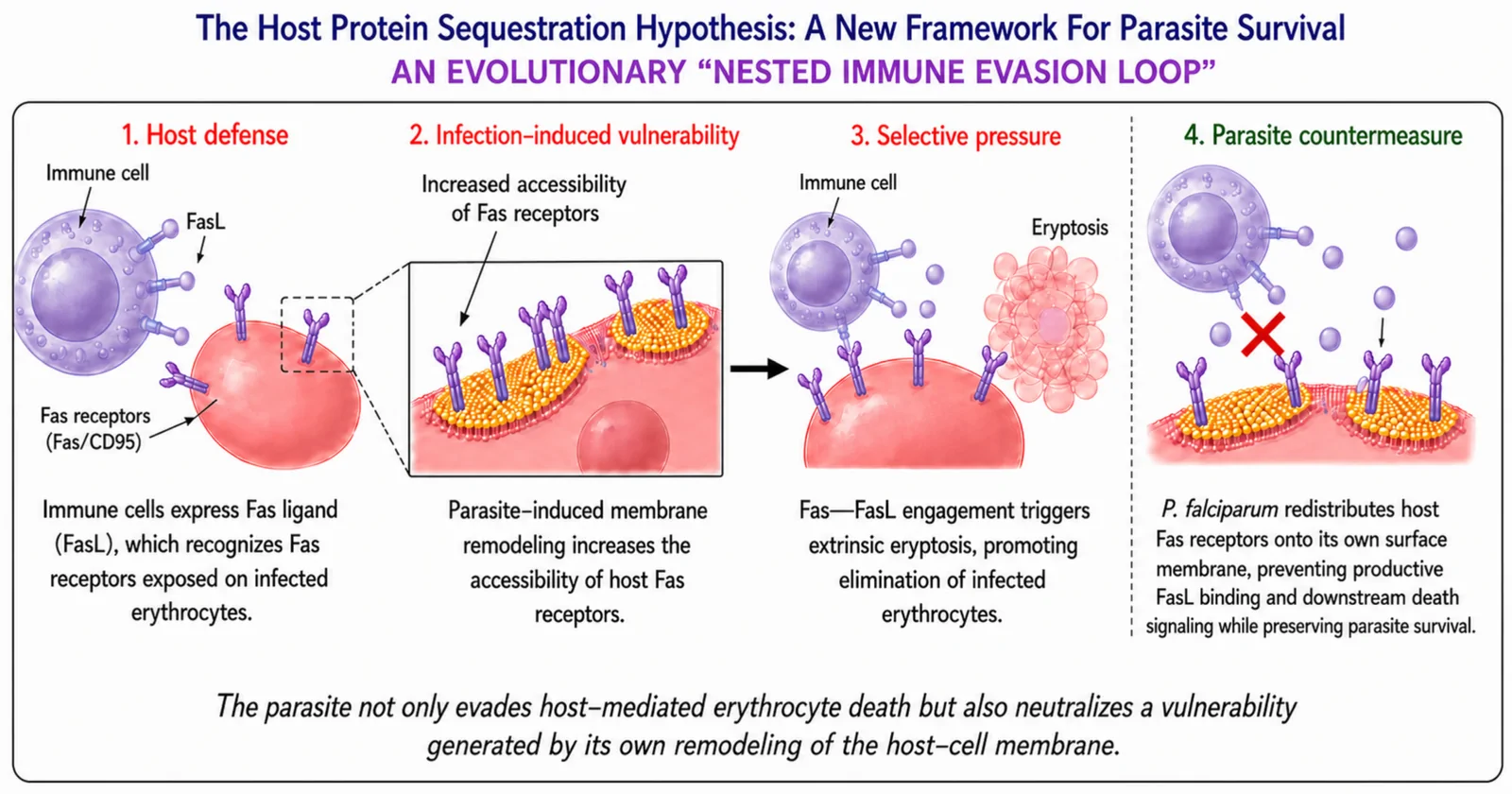
**Proposed host protein sequestration hypothesis as a nested immune-evasion mechanism in *Plasmodium falciparum*–infected erythrocytes.** Immune-cell–expressed Fas ligand (FasL) may recognize Fas/CD95 exposed on the erythrocyte surface, potentially promoting extrinsic eryptosis and clearance of infected cells. Parasite-induced remodeling of the host membrane and lipid rafts may increase Fas/CD95 accessibility, thereby creating a selective pressure for the parasite. The model proposes that *P. falciparum* counteracts this vulnerability by redistributing or sequestering host Fas/CD95 within remodeled membrane domains, limiting productive Fas–FasL engagement and downstream death signaling. This putative mechanism could prolong infected-erythrocyte survival and facilitate completion of the intraerythrocytic developmental cycle. Dashed boxes and connecting lines indicate magnified membrane regions. This model is hypothetical and requires experimental validation. This figure was created using ChatGPT-5.6SOL and subsequently reviewed and edited by the authors.

Conceptually, this may constitute a nested immune evasion loop—a second-order immune evasion mechanism. The parasite not only suppresses host-mediated erythrocyte death but also eliminates a vulnerability generated by its own remodeling of the host cell. Such an iterative strategy would highlight the dynamic nature of the evolutionary arms race between *P. falciparum* and its human host (Figure 2).

This sequestration strategy must be considered within the broader context of oxidative stress and lipid peroxidation that characterizes *P. falciparum* infection. The parasite consumes host hemoglobin and generates ROS that continuously assault the erythrocyte membrane, producing 4-HNE and other reactive aldehydes [10,15]. These electrophilic species not only damage membrane proteins and lipids but also serve as potent signaling molecules that can trigger CASMER formation and eryptosis [16,18]. Thus, the parasite faces a dual challenge: it must detoxify ROS and 4-HNE to prevent oxidative damage to its own macromolecules, while also preventing these same oxidative signals from activating host eryptotic pathways. The sequestration of Fas into intracellular compartments may represent one component of a broader oxidative defense strategy, potentially working in concert with the import of host peroxiredoxin-2 [86] and the remodeling of membrane lipid composition to reduce susceptibility to lipid peroxidation. By physically removing Fas from the membrane, the parasite may effectively uncouple oxidative stress signals from their downstream eryptotic effectors, thereby raising the threshold at which oxidative damage triggers host cell clearance.

### 5.1. Sequestration of Endogenous Erythrocyte Proteins and Redox Defense

The intraerythrocytic survival of *P. falciparum* heavily depends on both the continuous consumption and the targeted functional sequestration of endogenous erythrocyte proteins [86]. While the parasite aggressively degrades host hemoglobin to procure essential amino acids, this catabolic process generates massive quantities of reactive oxygen species [9,10]. To neutralize this profound oxidative stress, *P. falciparum* actively imports human peroxiredoxin-2 from the host cytoplasm directly into its own cytosol, coupling it to the plasmodial thioredoxin-1 reducing system to create an indispensable core component of its redox defense network [86].

### 5.2. Molecular Camouflage Through Host Protein Acquisition

Beyond its essential role in nutrient acquisition and parasite growth, the *P. falciparum* exomembrane system provides a sophisticated mechanism to remodel the immunological identity of the infected erythrocyte. Through parasite-derived trafficking structures, including the tubovesicular network and Maurer’s clefts, *P. falciparum* can internalize and redistribute host-derived plasma proteins to the infected red blood cell surface [101]. Among these host molecules, vitronectin represents a critical example of molecular camouflage, as its targeted acquisition and surface exposure on infected erythrocytes has been associated with reduced recognition and engulfment by human macrophages. By decorating the parasite-modified erythrocyte membrane with a self-derived host protein, *P. falciparum* may partially mask parasite-associated molecular patterns and interfere with innate immune surveillance mechanisms. This strategy illustrates a broader principle of immune evasion in which pathogens exploit host-derived molecules to alter the “immunological identity” of infected cells and delay their clearance. In addition to vitronectin, the parasite can capture other host plasma components, such as plasminogen, which may be processed into angiostatin-like fragments capable of modulating host endothelial responses and contributing to a microenvironment favorable for parasite persistence [101]. Thus, the exomembrane system represents not only a nutrient acquisition pathway but also a platform for molecular mimicry and immune modulation, allowing *P. falciparum* to manipulate host recognition pathways while maintaining intracellular survival.

Beyond molecular camouflage through protein acquisition, the oxidative modification of membrane lipids by 4-HNE may indirectly modulate immune recognition. 4-HNE adduction to erythrocyte membrane proteins generates neo-antigenic epitopes that can be recognized by naturally occurring antibodies and complement components [102,103]. In the context of malaria, oxidative damage to the infected erythrocyte membrane could therefore enhance opsonization and phagocytic clearance, adding another layer of immune pressure that the parasite must evade. Conversely, the extensive vesiculation induced by oxidative stress and 4-HNE may serve to shed these damaged epitopes, effectively “cleaning” the erythrocyte surface and reducing its immunological visibility [85,94]. The parasite may exploit this vesiculation process to selectively remove 4-HNE-modified proteins and lipids from the host cell surface, thereby limiting the accumulation of damage-associated molecular patterns (DAMPs) that would otherwise promote immune recognition. This adds a third dimension to the Host Protein Sequestration Hypothesis: selective sequestration may be accompanied by selective shedding, with both mechanisms working in concert to maintain a permissive immunological surface on the infected erythrocyte.

### 5.3. Subversion of Host Survival Signaling and Modulation of Eryptosis Through Host-Protein Hijacking

Beyond molecular camouflage through the acquisition of host-derived proteins, *P. falciparum* regulates the fate of the infected erythrocyte by exploiting the residual signaling machinery and functional proteins retained by this highly specialized host cell. Although mature erythrocytes lack a nucleus, they preserve dynamic signaling networks involved in membrane organization, calcium homeostasis, metabolism, and stress responses [104,105]. By co-opting these host-derived pathways, the parasite remodels the erythrocyte physiology to maintain a permissive intracellular environment while delaying premature immune-mediated clearance.

Among the host components that may be affected during infection, erythrocyte kinases such as p21-activated kinase (PAK) and elements of the MAPK pathway, including MEK, are part of the altered phosphosignaling landscape induced by *P. falciparum* [60,104]. Although their direct contribution to specific eryptotic events remains incompletely defined, these pathways illustrate how the parasite exploits host regulatory networks rather than relying exclusively on parasite-encoded factors. Similarly, the host survival protein BCL-xL has been shown to be required for efficient parasite proliferation and to redistribute toward parasite-associated compartments during infection, further supporting the functional repurposing of erythrocyte proteins by the parasite [60].

This manipulation extends to eryptosis, the programmed death-like process responsible for the removal of damaged erythrocytes. Rather than completely suppressing eryptosis, *P. falciparum* appears to regulate the threshold between cellular stress and immune clearance. Infection induces oxidative stress, calcium dysregulation, and membrane alterations that can promote phosphatidylserine (PS) exposure, a key elimination signal recognized by phagocytes. Indeed, increased PS exposure in infected erythrocytes has been associated with elevated intracellular calcium and reduced membrane cholesterol, promoting recognition by monocytes [106]. However, parasite-mediated mechanisms, including PI3K-dependent regulation of PS externalization, may counteract excessive exposure and prolong the infected erythrocyte survival [107]. Thus, parasite survival depends on a dynamic equilibrium in which host–cell stress responses are not eliminated but temporally controlled.

The ability of *P. falciparum* to manipulate eryptosis signaling is further reflected in studies showing that pharmacological induction of eryptosis promotes PS exposure, membrane blebbing, and extracellular vesicle release from infected erythrocytes, linking host–cell death pathways with membrane remodeling [108]. Together, these findings support a model in which *P. falciparum* hijacks erythrocyte proteins and signaling networks to regulate host–cell fate throughout infection: maintaining viability during intracellular replication, limiting premature immune clearance, and ultimately coordinating controlled host–cell rupture for parasite dissemination.

### 5.4. Biomechanical Remodeling and Hijacking of Host Calpain-1 for Controlled Egress: Repurposing Host Stress Signals for Parasite Dissemination

Beyond manipulating immune recognition and delaying erythrocyte clearance, *P. falciparum* also exploits host biomechanical and proteolytic mechanisms to control the terminal stage of its intraerythrocytic developmental cycle. Successful parasite dissemination requires a highly coordinated rupture event involving the sequential disruption of the parasitophorous vacuole membrane and the surrounding erythrocyte plasma membrane, allowing the release of newly formed merozoites for subsequent invasion cycles [109,110]. Rather than relying exclusively on parasite-derived factors, *P. falciparum* co-opts host cellular machinery, including the calcium-dependent cysteine protease human calpain-1, to execute the final structural remodeling required for efficient erythrocyte rupture.

During schizont maturation, parasite-driven alterations in calcium homeostasis contribute to the activation of host calpain-1, which promotes the degradation of key components of the erythrocyte cytoskeletal network, thereby reducing membrane stability and facilitating merozoite release [109,110]. This process highlights the dual role of calcium signaling during malaria infection. In earlier stages of infection, excessive or sustained intracellular calcium elevation can activate eryptotic pathways, including Gardos channel activation, membrane asymmetry disruption, and phosphatidylserine (PS) exposure, ultimately promoting recognition and clearance of infected erythrocytes by phagocytic cells [61,106]. However, at the terminal schizont stage, *P. falciparum* appears to redirect calcium-dependent signaling toward a parasite-controlled biomechanical program, converting a potentially detrimental host–cell stress signal into a mechanism that promotes controlled cellular rupture.

The activation of calpain-1 therefore represents not simply a consequence of erythrocyte damage but a temporally regulated exploitation of host proteolytic machinery. By controlling the timing and extent of cytoskeletal degradation, the parasite ensures that erythrocyte destabilization occurs only after completion of intracellular replication, preventing premature loss of the host cell before merozoite maturation. Consistent with this process, infected erythrocytes approaching egress display marked biomechanical alterations, including increased non-Gaussian membrane fluctuations associated with mechanical stress generated by the expanding merozoite population within the host cell [110].

From an immune evasion perspective, this strategy illustrates that *P. falciparum* does not simply suppress host–cell death pathways; instead, it selectively rewires host–cell fate according to the requirements of its developmental cycle. During most of the intraerythrocytic phase, parasite survival depends on delaying premature eryptotic signals that would promote splenic removal. Conversely, once replication is complete, the parasite activates host-derived mechanisms that facilitate rapid erythrocyte rupture and merozoite dissemination. This stage-dependent manipulation of calcium signaling, protease activity, and membrane biomechanics allows *P. falciparum* to balance immune avoidance with efficient transmission, demonstrating how parasite survival depends on precise temporal control of host–cell physiology.

**Figure 3:**
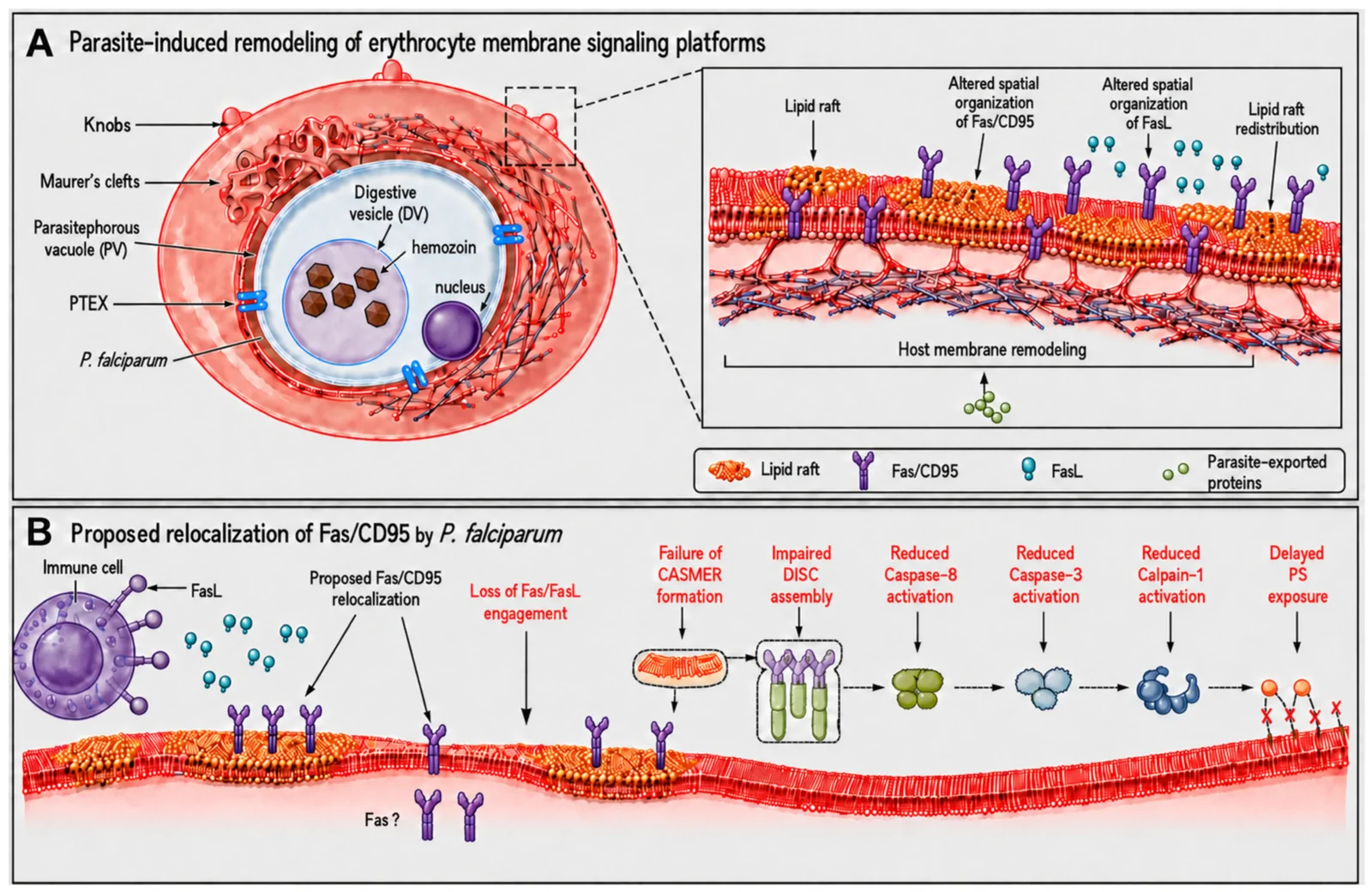
**Proposed remodeling of erythrocyte membrane signaling platforms and disruption of Fas/CD95-dependent eryptosis by *Plasmodium falciparum***. (**A**) Schematic representation of an infected erythrocyte showing the parasitophorous vacuole (PV), the Plasmodium translocon of exported proteins (PTEX), Maurer’s clefts, knobs, the digestive vesicle (DV), hemozoin, and the parasite nucleus. Parasite-exported proteins promote extensive remodeling of the host membrane and cytoskeleton, potentially altering lipid-raft distribution and the spatial organization of Fas/CD95 and extracellular Fas ligand (FasL). The boxed region is shown at higher magnification. (**B**) Proposed consequences of Fas/CD95 redistribution during infection. Reduced availability of Fas/CD95 at the erythrocyte surface may limit productive Fas–FasL engagement, impair CASMER formation and death-inducing signaling complex (DISC) assembly, and consequently reduce caspase-8, caspase-3, and calpain-1 activation. These alterations are predicted to delay phosphatidylserine (PS) exposure and the subsequent clearance of infected erythrocytes. The intracellular localization marked “Fas?” represents a hypothetical destination of relocalized Fas/CD95. This mechanistic model remains to be experimentally validated. This figure was created with ChatGPT (GPT-5.6SOL).

Figure 3 summarizes the proposed pathways used by *P. falciparum* to prevent the disruption of the infected erythrocyte before the culmination of its 48 h replication period inside the host red blood cell.

## 6. Future Directions and Challenges

The Host Protein Sequestration Hypothesis, while offering a cohesive and mechanistically plausible framework for *P. falciparum* immune evasion, particularly through the manipulation of host-derived proteins and erythrocyte signaling networks, rests predominantly on inferential evidence derived from indirect observations. As with any speculative model that bridges host cell biology and parasite effector function, several critical limitations and alternative interpretations must be explicitly acknowledged.

First, our model postulates specific physical interactions between parasite-encoded effector proteins and the host Fas receptor. Currently, there is a conspicuous absence of direct biochemical evidence for these interactions. Although Fas represents a relevant candidate because of its role in immune recognition and eryptosis-related signaling, this hypothesis should be considered an example within a broader model of selective host protein manipulation rather than the sole mechanism of parasite-mediated immune evasion. The hypothesis is built upon co-localization patterns within lipid rafts and the temporal correlation between protein export and membrane reorganization; however, co-localization does not equate to molecular binding [76]. We lack confirmatory data from classical protein–protein interaction assays, such as co-immunoprecipitation (Co-IP) from infected erythrocyte lysates, proximity ligation assays (PLA) to visualize in situ interactions, or surface plasmon resonance (SPR) to define binding affinities. It remains formally possible that these parasite proteins and host Fas occupy overlapping membrane territories without establishing a stable, functionally relevant complex. Furthermore, if such interactions do occur, they may be transient, of low affinity, or heavily dependent on the unique lipid microenvironment of the infected cell, making them notoriously difficult to capture using standard biochemical techniques.

Second, a major interpretative challenge lies in distinguishing a specific, targeted biological process from a passive epiphenomenon of global membrane destruction. During the 48 h intraerythrocytic cycle, *P. falciparum* unleashes a catastrophic wave of remodeling upon the host cell, dismantling the cytoskeleton, disrupting phospholipid asymmetry, and profoundly altering lipid packing and cholesterol distribution [25,26]. The redistribution of Fas into intracellular compartments or into atypical microdomains could theoretically be a non-specific physical consequence of this overall structural chaos, rather than the result of a finely tuned, evolutionarily selected “hijacking” pathway. This distinction is particularly important because infected erythrocytes naturally regulate membrane composition through mechanisms such as extracellular vesicle shedding, suggesting that loss of a surface protein does not necessarily indicate active parasite-mediated sequestration. If the erythrocyte membrane is mechanically stressed and deformed, surface proteins may passively aggregate, internalize, or shed without requiring active parasite-driven transport. Validating active specificity will require demonstrating that the sequestration of Fas occurs independently of, or disproportionately to, other abundant surface proteins (e.g., Band 3 or Glycophorin A).

Third, the experimental foundation for this hypothesis is derived almost exclusively from in vitro culture systems. While these systems are indispensable for dissecting molecular mechanisms, they represent a highly reductionist environment that fails to capture the dynamic physiological complexity of human malaria. In vivo, infected erythrocytes are subjected to continuous hemodynamic shear stress, splenic filtration forces, fluctuating febrile temperatures, and a diverse array of immune effector cells (including macrophages and NK cells). The kinetics of eryptosis, the bioavailability of Fas Ligand, and the efficiency of splenic clearance are profoundly different in circulation compared to static culture flasks. Therefore, the extent to which parasite-mediated Fas sequestration actually delays premature clearance in a living host remains entirely speculative. Future studies should therefore evaluate not only Fas localization but also the broader consequences of host protein redistribution on erythrocyte survival, immune recognition, and parasite fitness. For instance, an in vitro delay of a few hours in phosphatidylserine exposure might be biologically irrelevant in the context of a rapid splenic passage. As such, the present model urgently requires validation in physiologically relevant animal models—such as an infection with *P. berghei* or the use of a humanized mouse model—and, ideally, verification using primary patient isolates, where the interplay of host genetic variation and parasite strain diversity can be assessed.

Lastly, and perhaps most critically, there is a fundamental difficulty in experimentally discriminating between active sequestration by the parasite and passive degradation or disposal by the host erythrocyte itself. Mature erythrocytes, despite lacking internal organelles, possess a well-documented capacity to extrude damaged membrane components via the shedding of extracellular microvesicles [85,94]. This outward-facing clearance mechanism naturally removes Fas receptors from the cell surface as a homeostatic response to aging or oxidative stress. This shedding process effectively extrudes membrane-bound proteins, including the FAS receptor, into the extracellular space rather than pulling them inward into the cytoplasm. Crucially, the release of death receptors on membrane-derived vesicles is a well-established physiological phenomenon; indeed, both biologically active FAS antigen and its cognate ligand, FASL, are known to be exfoliated from cell surfaces on plasma membrane-derived extracellular vesicles in a fully bioactive configuration [111]. Therefore, the disappearance of Fas from the erythrocyte surface should not be interpreted by itself as evidence of parasite-driven internalization or functional neutralization. Alternatively, the parasite could actively degrade Fas within the host cytosol or the parasitophorous vacuole using exported proteases. The disappearance of Fas from the erythrocyte surface does not inherently prove that the parasite has “pulled it inward” for functional neutralization. To differentiate these scenarios, future studies must deploy quantitative live-cell imaging combined with compartment-specific biosensors to trace the real-time fate of labeled Fas, alongside comprehensive proteomic analyses of both parasite-derived compartments and released microvesicles.

Despite these substantial caveats, we emphasize that none of these limitations invalidate the core premise of the hypothesis. Rather, they sharply define the experimental boundaries that must be crossed to elevate the model from a conceptual framework to a validated biological mechanism. Importantly, validation of this hypothesis does not require that Fas represent the only host protein manipulated by the parasite, but rather that *P. falciparum* selectively exploits host molecular components to regulate erythrocyte fate. The hypothesis provides a clearly articulated, testable series of predictions—ranging from specific protein interactions to functional rescue experiments—that now serve as a structured guide for future research into host-directed antimalarial interventions.

The Host Protein Sequestration Hypothesis makes several testable predictions: (1) parasite-encoded lipid-raft scaffolding proteins will physically interact with host Fas; and potentially with additional host proteins involved in erythrocyte survival, membrane remodeling, or immune recognition; (2) Fas and other selected host proteins will localize to intracellular parasite-induced compartments (Maurer’s clefts, parasitophorous vacuole) in infected erythrocytes, but not in uninfected controls; (3) disruption of these interactions (via genetic knockout or pharmacological inhibition) will restore Fas surface expression or alter host–cell susceptibility to immune-mediated clearance; (4) the sequestration phenomenon will correlate temporarilly with the parasite developmental stage.

As is clear, this hypothesis awaits experimental confirmation.

## 7. Conclusions

The intraerythrocytic survival of *P. falciparum* depends on a delicate balance between exploiting the host erythrocyte for nutrients and maintaining its structural viability. The parasite must manage the profound oxidative stress generated by its own metabolism to prevent premature host cell clearance via eryptosis while also avoiding its own stress-induced cell death.

To achieve this, *P. falciparum* extensively remodels the host membrane and hijacks erythrocyte proteins and signaling pathways to modulate the threshold for eryptosis activation. We propose the Host Protein Sequestration Hypothesis, which posits that the parasite actively internalizes host death receptors, such as Fas, from the host surface to prevent engagement with FasL from immune cells. This targeted manipulation, potentially involving parasite lipid-raft scaffolding proteins, ensures the infected erythrocyte remains in circulation long enough to complete the parasite’s replication cycle.

Understanding these precise host–parasite interactions offer a pathway for novel host-directed therapies. Disrupting parasite-induced membrane remodeling or restoring immune recognition mechanisms could selectively promote the premature clearance of infected cells. Ultimately, the parasite’s success depends on its ability to transform the erythrocyte into a viable, immunologically altered niche. Elucidating how it hijacks host proteins and coordinates survival with dissemination will be essential for identifying new therapeutic vulnerabilities and defining host-directed strategies against malaria.

## Figures and Tables

**Table 1:** Phenotypic Evidence and Molecular Mechanisms of Regulated Cell Death in *Plasmodium falciparum*.

Marker	Detection Method	Observed in *P. falciparum*?	Limitations/Specificity
Chromatin condensation	Hoechst, DAPI, acridine orange	Yes (under drug-induced or oxidative stress)	May overlap with autophagy or necrosis
DNA fragmentation	TUNEL	Yes	Detects general genomic damage; not specific to apoptosis
Loss of ΔΨm (mitochondrial membrane potential)	JC-1, TMRE, rhodamine 123	Yes (early event)	May also occur during severe necrosis
Caspase-like activity	CaspACE, CaspaTag	Yes (detected)	Probes are designed for caspases; may cross-react with metacaspases and other cysteine proteases
Phosphatidylserine (PS) externalization	Annexin V	Yes (in the host erythrocyte)	Difficult to distinguish host eryptosis from parasite apoptosis
Cell shrinkage/vacuolization	Microscopy	Yes	Not specific to apoptosis
Apoptotic bodies	Electron microscopy	Not confirmed	

## Data Availability

No new data were created or analyzed in this study. Data sharing is not applicable to this article.

## References

[1] Hayton K., Su X. (2008). Drug resistance and genetic mapping in *Plasmodium falciparum*. Curr. Genet..

[2] Blasco B., Leroy D., Fidock D.A. (2017). Antimalarial drug resistance: Linking *Plasmodium falciparum* parasite biology to the clinic. Nat. Med..

[3] World Health Organization (2025). World Malaria Report 2025: Addressing the Threat of Antimalarial Drug Resistance.

[4] Habarugira F., Nsabimana A., Uwimana A., Mutesa L. (2026). The Global Landscape of *Plasmodium falciparum* Drug Resistance Markers, 2005–2025: A Systematic Review and Meta-Analysis. Pathogens.

[5] Ralph S.A., van Dooren G.G., Waller R.F., McFadden G.I. (2004). Metabolic maps and functions of the *Plasmodium falciparum* apicoplast. Nat. Rev. Microbiol..

[6] Bonive-Boscan A.D., Acosta H., Rojas A. (2024). Metabolic changes that allow *Plasmodium falciparum* artemisinin-resistant parasites to tolerate oxidative stress. Front. Parasitol..

[7] Gonçalves A.F., Lima-Pinheiro A., Ferreira P.E. (2024). Ubiquitin-proteasome system in *Plasmodium*: A potential antimalarial target to overcome resistance–A systematic review. Front. Med..

[8] Ouji M., Augereau J.M., Paloque L., Benoit-Vical F. (2024). In artemisinin-resistant falciparum malaria parasites, mitochondrial metabolic pathways are essential for survival but not those of apicoplast. Int. J. Parasitol. Drugs Drug Resist..

[9] Kumar S., Bandyopadhyay U. (2005). Free heme toxicity and its detoxification systems in human. Toxicol. Lett..

[10] Percário S., Moreira D.R., Gomes B.A.Q., Ferreira M.E.S., Gonçalves A.C.M., Laurindo P.S.O., Vilhena T.C., Dolabela M.F., Green M.D. (2012). Oxidative Stress in Malaria. Int. J. Mol. Sci..

[11] Sun H., Zhang Y., Wang J., Li X., Chen L. (2024). Dobinin K Displays Antiplasmodial Activity through Disruption of *Plasmodium falciparum* Mitochondria and Generation of Reactive Oxygen Species. Molecules.

[12] Esterbauer H., Schaur R.J., Zollner H. (1991). Chemistry and biochemistry of 4-hydroxynonenal, malonaldehyde and related aldehydes. Free Radic. Biol. Med..

[13] Ayala A., Muñoz M.F., Argüelles S. (2014). Lipid Peroxidation: Production, Metabolism, Biological Signaling and Mechanisms of Toxic Action. Oxid. Med. Cell. Longev..

[14] Poli G., Schaur R.J., Siems W.G., Leonarduzzi G. (2008). 4-Hydroxynonenal: A Membrane Lipid Oxidation Product of Medicinal Interest. Med. Res. Rev..

[15] Schaur R.J., Siems W., Bresgen N., Eckl P.M. (2015). 4-Hydroxynonenal as a Bioactive Product of Lipid Peroxidation. Int. J. Mol. Sci..

[16] Lang F., Lang K.S., Lang P.A., Huber S.M., Wieder T. (2003). Mechanisms and Significance of Eryptosis. Antioxid. Redox Signal..

[17] Nicolay J.P., Gatz S., Liebig G., Gulbins E., Lang F. (2006). Stimulation of Suicidal Erythrocyte Death by Methylglyoxal. Cell. Physiol. Biochem..

[18] Mollinedo F., Gajate C. (2022). Clusters of apoptotic signaling molecule-enriched rafts, CASMERs: Membrane platforms for protein assembly in Fas/CD95 signaling and targets in cancer therapy. Biochem. Soc. Trans..

[19] Meslin B., Beurchon C., Weinberg M., Tibayrenc M., Koella J.C. (2007). Features of apoptosis in *Plasmodium falciparum* erythrocytic stage through a putative role of PfMCA1 metacaspase-like protein. J. Infect. Dis..

[20] Mutai B.K., Waitumbi J.N. (2010). Apoptosis stalks *Plasmodium falciparum* maintained in continuous culture condition. Malar. J..

[21] Jiménez-Ruiz A., Alzate O., Ruiz-Arguello M.B., Alonso C. (2010). Apoptotic markers in protozoan parasites. Parasit. Vectors.

[22] Smirlis D., Bisti W. (2010). Targetting essential pathways in trypanosomatids gives insights into protozoan mechanisms of cell death. Parasit. Vectors.

[23] Proto W.R., Coombs G.H., Mottram J.C. (2013). Cell death in parasitic protozoa: Regulated or incidental?. Nat. Rev. Microbiol..

[24] Obeng E. (2021). Apoptosis (programmed cell death) and its signals—A review. Braz. J. Biol..

[25] Hsiao L.L., Howard R.J., Aikawa M., Taraschi T.F. (1991). Modification of host cell membrane lipid composition by the intra-erythrocytic human malaria parasite *Plasmodium falciparum*. Biochem. J..

[26] Spillman N.J., Beck J.R., Goldberg D.E. (2015). Protein export into malaria parasite–infected erythrocytes: Mechanisms and functional consequences. Annu. Rev. Biochem..

[27] Alves-Rosa M.F., Tayler N.M., Dorta D., Coronado L.M., Spadafora C.P. (2024). *P. falciparum* Invasion and Erythrocyte Aging. Cells.

[28] An X., Mohandas N. (2008). Disorders of red cell membrane. Br. J. Haematol..

[29] Mohandas N., Gallagher P.G. (2008). Red cell membrane: Past, present, and future. Blood.

[30] Goodman S.R., Kurdia A., Ammann L., Kakhniashvili D., Daescu O. (2007). The human red blood cell proteome and interactome. Exp. Biol. Med..

[31] Mohandas N., An X. (2012). Malaria and human red blood cells. Med. Microbiol. Immunol..

[32] Shen S., Shao Y., Li C. (2023). Different types of cell death and their shift in shaping disease. Cell Death Discov..

[33] Liu S., Yao S., Yang H., Liu S., Wang Y., Zhang Y. (2023). Autophagy: Regulator of cell death. Cell Death Dis..

[34] Glick D., Barth S., Macleod K.F. (2010). Autophagy: Cellular and molecular mechanisms. J. Pathol..

[35] Chao D.T., Korsmeyer S.J. (1998). BCL-2 family: Regulators of cell death. Annu. Rev. Immunol..

[36] Wei M.C., Zong W.X., Degenhardt E.H., Lindsten T., Thompson C.B., Korsmeyer S.J. (2001). Proapoptotic BAX and BAK: A requisite gateway to mitochondrial dysfunction and death. Science.

[37] Galluzzi L., Bravo-San Pedro J.M., Vitale I., Aaronson S.A., Abrams J.M., Adam D., Alnemri E.S., Altucci L., Andrews D., Annicchiarico-Petruzzelli M. (2015). Essential versus accessory aspects of cell death: Recommendations of the NCCD 2015. Cell Death Differ..

[38] Kulkarni M., Hardwick J.M. (2023). Programmed cell death in unicellular versus multicellular organisms. Annu. Rev. Genet..

[39] Rathore S., Jain S., Sinha D., Saha S., Asad M., Sharma A., Habib S., Mohmmed A. (2011). Disruption of a mitochondrial protease machinery in *Plasmodium falciparum* is an intrinsic signal for parasite cell death. Cell Death Dis..

[40] Pandey R.K., Sharma S., Singh S., Rathore S., Mohmmed A. (2007). 4-Hydroxynonenal Production in Malaria-Infected Erythrocytes and Its Role in Oxidative Stress. Free Radic. Res..

[41] Kroemer G., Galluzzi L., Vandenabeele P., Abrams J., Alnemri E.S., Baehrecke E.H., Blagosklonny M.V., El-Deiry W.S., Golstein P., Green D.R. (2009). Classification of cell death: Recommendations of the Nomenclature Committee on Cell Death 2009. Cell Death Differ..

[42] Kaczanowski S., Sajid M., Reece S.E. (2011). Evolution of apoptosis-like programmed cell death in unicellular protozoan parasites. Parasit. Vectors.

[43] Reece S.E., Pollitt L.C., Colegrave N., Gardner A. (2011). The meaning of death: Evolution and ecology of apoptosis in protozoan parasites. PLoS Pathog..

[44] Reece S.E., Drew D.R., Gardner A. (2008). Sex ratio adjustment and kin discrimination in malaria parasites. Nature.

[45] Paz-y-Miño-C G., Espinosa A. (2016). Kin Discrimination in Protists: From Many Cells to Single Cells and Backwards. J. Eukaryot. Microbiol..

[46] Correa R., Dorta D., Pineda L., Padmore P., Spadafora C., Coronado L.M. (2019). Extracellular vesicles carrying lactate dehydrogenase induce suicide in increased population density of *Plasmodium falciparum* in vitro. Sci. Rep..

[47] Engelbrecht D., Durand P.M., Coetzer T.L. (2012). On programmed cell death in *Plasmodium falciparum*: Status quo. J. Trop. Med..

[48] Gardner M.J., Hall N., Fung E., White O., Berriman M., Hyman R.W., Carlton J.M., Pain A., Nelson K.E., Bowman S. (2002). Genome sequence of the human malaria parasite *Plasmodium falciparum*. Nature.

[49] Vieira W.A., Coetzer T.L. (2016). Localization and interactions of *Plasmodium falciparum* SWIB/MDM2 homologues. Malar. J..

[50] Feder M.E., Hofmann G.E. (1999). Heat-shock proteins, molecular chaperones, and the stress response: Evolutionary and ecological physiology. Annu. Rev. Physiol..

[51] Oduro-Kwateng E., Owusu K.B., Amoah L.E. (2025). Computational Analysis of *Plasmodium falciparum* DNA Damage Inducible Protein 1 (PfDdi1): Insights into Binding of Artemisinin and its Derivatives and Implications for Antimalarial Drug Design. Cell Biochem. Biophys..

[52] Galluzzi L., Vitale I., Aaronson S.A., Abrams J.M., Adam D., Agostinis P., Alnemri E.S., Altucci L., Amelio I., Andrews D.W. (2018). Molecular mechanisms of cell death: Recommendations of the Nomenclature Committee on Cell Death 2018. Cell Death Differ..

[53] Tanneru N., Srivastava A., Kumar A., Sharma R., Habib S. (2023). *Plasmodium* DDI1 is a potential therapeutic target and important chromatin-associated protein. Int. J. Parasitol..

[54] Loo D.T., Didenko V.V. (2011). In Situ Detection of Apoptosis by the TUNEL Assay: An Overview of Techniques. DNA Damage Detection In Situ, Ex Vivo, and In Vivo: Methods and Protocols.

[55] Mirzayans R., Murray D. (2020). Do TUNEL and other apoptosis assays detect cell death in preclinical studies?. Int. J. Mol. Sci..

[56] Vercammen D., van de Cotte B., De Jaeger G., De Rycke R., Inzé D., Van Breusegem F. (2004). Type II Metacaspases Atmc4 and Atmc9 of *Arabidopsis thaliana* Cleave Substrates after Arginine and Lysine. J. Biol. Chem..

[57] Ch’Ng J.H., Liew K., Schiefner A., Chan F.K., Tan K.S. (2010). A programmed cell death pathway in the malaria parasite *Plasmodium falciparum* has general features of mammalian apoptosis but is mediated by clan CA cysteine proteases. Cell Death Dis..

[58] Tsiatsiani L., Van Breusegem F., Gallois P., Zavialov A., Lam E., Bozhkov P.V. (2011). Metacaspases. Cell Death Differ..

[59] Alzoubi K., Alktifan B., Oswald G., Fezai M., Abed M., Lang F. (2014). Breakdown of Phosphatidylserine Asymmetry Following Treatment of Erythrocytes with Lumefantrine. Toxins.

[60] Boulet C., Doerig C.D., Carvalho T.G. (2018). Manipulating Eryptosis of Human Red Blood Cells: A Novel Antimalarial Strategy?. Front. Cell. Infect. Microbiol..

[61] Scovino A.M., Totino P.R.R., Morrot A. (2022). Eryptosis as a New Insight in Malaria Pathogenesis. Front. Immunol..

[62] Kumar B., Chaubey S., Shah P., Tanveer A., Madambat S., Siddiqi M.I., Habib S. (2019). Metacaspase-3 of *Plasmodium falciparum*: An atypical trypsin-like serine protease. Int. J. Biol. Macromol..

[63] Callan-Jones A., Al-Kilani A.M., Eckly A., Heussler V., Schnalke P., Knuepfer E., Lagache T., Fleury V., Baum J. (2012). Red Blood Cell Membrane Dynamics during Malaria Parasite Egress. Biophys. J..

[64] Tkachenko A., Havranek O. (2025). Cell death signaling in human erythron: Erythrocytes lose the complexity of cell death machinery upon maturation. Apoptosis.

[65] Lang F., Qadri S.M. (2012). Mechanisms and Significance of Eryptosis, the Suicidal Death of Erythrocytes. Blood Purif..

[66] de Koning-Ward T.F., Gilson P.R., Boddey J.A., Rug M., Smith B.J., Papenfuss A.T., Sanders P.R., Lundie R.J., Maier A.G., Cowman A.F. (2009). A newly discovered protein export machine in malaria parasites. Nature.

[67] Maier A.G., Rug M., O’Neill M.T., Brown M., Chakravorty S., Szestak T., Chesson J., Wu Y., Hughes K., Coppel R.L. (2009). Malaria parasite proteins that remodel the host erythrocyte. Nat. Rev. Microbiol..

[68] Sam-Yellowe T.Y. (2009). The role of the Maurer’s clefts in protein transport in *Plasmodium falciparum*. Trends Parasitol..

[69] Mundwiler-Pachlatko E., Beck H.-P. (2013). Maurer’s clefts, the enigma of *Plasmodium falciparum*. Proc. Natl. Acad. Sci. USA.

[70] Lau C.K.Y., Turner L., Jespersen J.S., Lowe E.D., Petersen B., Wang C.W., Petersen J.E., Lusingu J., Theander T.G., Lavstsen T. (2015). Structural Conservation Despite Huge Sequence Diversity Allows EPCR Binding by the PfEMP1 Family Implicated in Severe Childhood Malaria. Cell Host Microbe.

[71] Craig A., Scherf A. (2001). Molecules on the surface of the *Plasmodium falciparum* infected erythrocyte and their role in malaria pathogenesis and immune evasion. Mol. Biochem. Parasitol..

[72] Scherf A., Lopez-Rubio J.J., Riviere L. (2008). Antigenic Variation in *Plasmodium falciparum*. Annu. Rev. Microbiol..

[73] Turner L., Lavstsen T., Berger S.S., Wang C.W., Petersen J.E., Avril M., Brazier A.J., Freeth J., Jespersen J.S., Nielsen M.A. (2013). Severe malaria is associated with parasite binding to endothelial protein C receptor. Nature.

[74] Dinko B., Pradel G. (2016). Immune evasion by *Plasmodium falciparum* parasites: Converting a host protection mechanism for the parasite’s benefit. Front. Microbiol..

[75] Scheel-Toellner D., Wang K., Craddock R., Webb P.R., McGettrick H.M., Assi L.K., Parkes N., Clough L.E., Gulbins E., Lord J.M. (2002). The death-inducing signalling complex is recruited to lipid rafts in Fas-induced apoptosis. Biochem. Biophys. Res. Commun..

[76] Gajate C., Gonzalez-Camacho F., Mollinedo F. (2009). Lipid raft connection between extrinsic and intrinsic apoptotic pathways. Biochem. Biophys. Res. Commun..

[77] Samuel B.U., Mohandas N., Harrison T., Hiller N.L., Haldar K. (2001). The Role of Cholesterol and Glycosylphosphatidylinositol-anchored Proteins of Erythrocyte Rafts in Regulating Raft Protein Content and Malarial Infection. J. Biol. Chem..

[78] Murphy S.C., Samuel B.U., Harrison T., Speicher K.D., Speicher D.W., Reid M.E., Prohaska R., Low P.S., Tanner M.J., Mohandas N. (2004). Erythrocyte detergent-resistant membrane proteins: Their characterization and selective uptake during malarial infection. Blood.

[79] Murphy S.C., Harrison T., Haldar K. (2006). Lipid rafts and malaria parasite infection of erythrocytes. Mol. Membr. Biol..

[80] Vallese F., Kim K., Ruiz A., Sanchez R., Perez C. (2026). Stomatin encapsulates aquaporin-1 and urea transporter–B in the erythrocyte membrane. Sci. Adv..

[81] Haile M.T., Smith A.B., Jones C.D., Wang X., Taylor R. (2026). Structural basis for host membrane binding and remodeling by invading malaria parasites. Cell.

[82] Lauer S., VanWye J., Harrison T., McManus H., Samuel B.U., Hiller N.L., Mohandas N., Haldar K. (2000). Vacuolar uptake of host components, and a role for cholesterol and sphingomyelin in malarial infection. EMBO J..

[83] Camacho P. (2003). Malaria parasites solve the problem of a low calcium environment. J. Cell Biol..

[84] Mandal D., Mazumder A., Das P., Kundu M., Basu J. (2005). Fas-, Caspase 8-, and Caspase 3-dependent Signaling Regulates the Activity of the Aminophospholipid Translocase and Phosphatidylserine Externalization in Human Erythrocytes. J. Biol. Chem..

[85] Kriebardis A.G., Antonelou M.H., Stamoulis K.E., Economou-Petersen E., Margaritis L.H., Papassideri I.S. (2008). RBC-derived vesicles during storage: Ultrastructure, protein composition, oxidation, and signaling components. Transfusion.

[86] Brizuela M., Huang H.M., Smith C., Burgio G., Foote S.J., McMorran B.J. (2014). Treatment of Erythrocytes with the 2-Cys Peroxiredoxin Inhibitor, Conoidin A, Prevents the Growth of *Plasmodium falciparum* and Enhances Parasite Sensitivity to Chloroquine. PLoS ONE.

[87] Bissinger R., Barklage E., Bissinger F., Föller M., Lang F. (2014). Macrophage Colony-Stimulating Factor-Induced Eryptosis. Cell. Physiol. Biochem..

[88] De Maria R., Zeuner A., Eramo A., Domenichelli C., Bonci D., Grignani F., Srinivasula S.M., Alnemri E.S., Testi R., Peschle C. (1999). Negative regulation of erythropoiesis by caspase-mediated cleavage of GATA-1. Nature.

[89] Testa U. (2004). Apoptotic mechanisms in the control of erythropoiesis. Leukemia.

[90] Cervantes S., Bunnik E.M., Saraf A., Conner M.B., Florens L., Le Roch K.G. (2014). The multifunctional autophagy pathway in the human malaria parasite, *Plasmodium falciparum*. Autophagy.

[91] Hain A.U.P., Bosch J. (2013). Autophagy in *Plasmodium*, A Multifunctional Pathway?. Comput. Struct. Biotechnol. J..

[92] Walczak M., Ganesan S.M., Niles J.C., Yeh E. (2018). ATG8 Is Essential Specifically for an Autophagy-Independent Function in Apicoplast Biogenesis in Blood-Stage Malaria Parasites. mBio.

[93] Johnstone R.M., Adam M., Hammond J.R., Orr L., Turbide C. (1987). Vesicle formation during reticulocyte maturation. Association of plasma membrane activities with released vesicles (exosomes). J. Biol. Chem..

[94] Larson M.C., Hillery C.A., Hogg N. (2014). Circulating membrane-derived microvesicles in redox biology. Free Radic. Biol. Med..

[95] Paul A., Ramdani G., Tatu U., Mantu K., Ray S. (2019). Studying the rigidity of red blood cells induced by *Plasmodium falciparum* infection. Sci. Rep..

[96] Dorta D., Padmore P., Correa R., Pineda L., Spadafora C., Sarmiento-Gómez E., Coronado L.M. (2024). Optical tweezers to measure the elasticity of red blood cells: A tool to study the erythrocyte response to antimalarials. Front. Malar..

[97] Cranston H.A., Boylan C.W., Carroll G.L., Sutera S.P., Williamson J.R., Gluzman I.Y., Krogstad D.J. (1984). *Plasmodium falciparum* maturation abolishes physiologic red cell deformability. Science.

[98] Mohan K., Ganguly N.K., Dubey M.L., Joshi W.K., Mahajan R.C. (1992). Oxidative damage of erythrocytes infected with *Plasmodium falciparum*. Ann. Hematol..

[99] Mavoungou E., Luty A.J.F., Kremsner P.G. (2003). Natural killer (NK) cell-mediated cytolysis of *Plasmodium falciparum*-infected human red blood cells in vitro. Eur. Cytokine Netw..

[100] Kurtzhals J.A., Reimert C.M., Tette E., Dunyo S.K., Koram K.A., Akanmori B.D., Nkrumah F.K., Hviid L. (1998). Increased eosinophil activity in acute *Plasmodium falciparum* infection--association with cerebral malaria. Clin. Exp. Immunol..

[101] El Chamy Maluf S., Bisson-Filho A.W., Oliveira R.C., Santos S.A., Thiemann O.H. (2020). Human plasma plasminogen internalization route in *Plasmodium falciparum*-infected erythrocytes. Malar. J..

[102] Weismann D., Hartvigsen K., Lauer N., Bennett K.L., Scholl H.P.N., Charbel Issa P., Cano M., Brandstätter H., Tsimikas S., Skerka C. (2011). Complement Factor H Binds Malondialdehyde Epitopes and Protects from Oxidative Stress. Nature.

[103] Hsu C.G., Chávez C.L., Zhang C., Sowden M., Yan C., Berk B.C. (2022). The lipid peroxidation product 4-hydroxynonenal inhibits NLRP3 inflammasome activation and macrophage pyroptosis. Cell Death Differ..

[104] Adderley J.D., John von Freyend S., Jackson S.A., Bird M.J., Burns A.L., Anar B., Metcalf T., Semblat J.-P., Billker O., Wilson D.W. (2020). Analysis of erythrocyte signalling pathways during *Plasmodium falciparum* infection identifies targets for host-directed antimalarial intervention. Nat. Commun..

[105] Yong J.J.M., Gao X., Prakash P., Ang J.W., Lai S.K., Chen M.W., Neo J.J.L., Lescar J., Li H.Y., Preiser P.R. (2024). Red blood cell signaling is functionally conserved in *Plasmodium* invasion. iScience.

[106] Fraser M., Jing W., Bröer S., Kurth F., Sander L.-E., Matuschewski K., Maier A.G. (2021). Breakdown in membrane asymmetry regulation leads to monocyte recognition of *Plasmodium falciparum*-infected red blood cells. PLoS Pathog..

[107] Zheng K., Li Q., Jiang N., Zhang X., Wang H. (2026). *Plasmodium* PI3K suppresses the externalization of phosphatidylserine on infected erythrocytes. Immun. Inflamm..

[108] Carrera-Bravo C., Zhou T., Chu T., Hang J.W., Modh H., Huang C., Zhang S., Hao H., Cabada-García M.J., Malleret B. (2025). Chloroquine induces eryptosis in *P. falciparum*-infected red blood cells and the release of extracellular vesicles with a unique protein profile. Front. Cell. Infect. Microbiol..

[109] Chandramohanadas R., Davis P.H., Beiting D.P., Harbut M.B., Darling C., Velmourougane G., Greenspan N.S., Ward G.E., Greenbaum D.C. (2009). Apicomplexan parasites co-opt host calpains to facilitate their escape from infected cells. Science.

[110] Chandramohanadas R., Park Y., Lui L., Li A., Quinn D., Liew K., Diez-Silva M., Sung Y., Dao M., Lim C.T. (2011). Biophysics of malarial parasite exit from infected erythrocytes. PLoS ONE.

[111] Albanese J., Meterissian S., Kontogiannea M., Dubreuil C., Hand A., Sorba S., Dainiak N. (1998). Biologically active Fas antigen and its cognate ligand are expressed on plasma membrane-derived extracellular vesicles. Blood.

